# Reply to McArdle and Austin, “Further detail required to assess additional evidence on agreement between VITEK 2 and broth microdilution piperacillin/tazobactam testing in cephalosporin resistant Enterobacterales”

**DOI:** 10.1128/jcm.00338-26

**Published:** 2026-05-14

**Authors:** Sandra S. Richter, Shawn H. MacVane

**Affiliations:** 1 Department of Laboratory Medicine and Pathology, Mayo Clinic 156400 , Jacksonville, Florida, USA; 2 bioMerieux, Inc., Hazelwood, Missouri, USA; Mayo Clinic, Rochester, Minnesota, USA

## REPLY

The primary objective of our previously published study was to compare the workflow and accuracy of two commercial antimicrobial susceptibility test (AST) systems (VITEK 2 and MicroScan) using a well-characterized collection of 200 Enterobacterales isolates tested by broth microdilution (BMD) as the reference standard, applying updated Clinical and Laboratory Standards Institute (CLSI) breakpoints (1). The study did not focus on ceftriaxone non-susceptible Enterobacterales isolates, as suggested in the initial sentence of the letter submitted by McArdle and Austin (2). Instead, the isolates for our study were selected to maximize a wide representation of contemporary β-lactam resistance genotypes (*n* = 106), as well as wild-type strains (*n* = 94). This is similar to the FDA recommendation of a 50% susceptible, 50% resistant phenotype distribution in data packages submitted by AST device manufacturers seeking 510K market clearance for an antimicrobial agent (3).

All 200 isolates were provided by Jones Microbiology Institute (JMI) and originated from the U.S. and Latin American medical centers enrolled in the SENTRY Antimicrobial Surveillance Program (4). A more detailed query to JMI regarding the source of the isolates revealed that none were from the CDC/FDA Antimicrobial Resistant Isolate Bank, and the span of SENTRY surveillance periods was 2015–2020. Across the study, we evaluated 22 antimicrobial agents. Importantly, isolates for our study were not selected on the basis of minimal inhibitory concentration (MIC) distributions for any of these agents, and no attempt was made to omit isolates with MICs close to the breakpoint.

To address the concerns raised in the letter, we provide the reference BMD MIC distributions for the two agents (piperacillin-tazobactam and ceftriaxone) mentioned in the letter (2) in Table 1 below, along with MIC distributions from the earliest time period available (2020) from the SENTRY public database for comparison (https://sentry-mvp.jmilabs.com/). Compared to the SENTRY surveillance data (*n* > 6,000 isolates), the MIC distribution in our 200 isolate challenge set shows a higher proportion of ceftriaxone and piperacillin-tazobactam non-susceptible isolates. This is expected and reflects the intentional enrichment of contemporary β-lactam resistance genotypes to ensure adequate challenge of AST system performance. Importantly, despite this enrichment, our previously published study maintains similar proportions of borderline piperacillin-tazobactam MICs at 8, 16, and 32 µg/mL and includes isolates across the full susceptible range, supporting its suitability as a balanced, yet resistance-focused challenge set.

**TABLE 1:** Reference broth microdilution MIC distributions

No. (%) of isolates with piperacillin-tazobactam MIC (µg/mL)										
	≤0.25	0.5	1	2	4	8	16	32	64	>64
Reference 1 (*n* = 200)	11 (5.5)	3 (1.5)	16 (8.0)	65 (32.5)	30 (15.0)	9 (4.5)	10 (5.0)	2 (1.0)	2 (1.0)	52 (26.0)
Sentry 2020^*a*^ (*n* = 6,126)	326 (5.3)	267 (4.4)	815 (13.3)	2,437 (39.8)	1,095 (17.9)	367 (6.0)	217 (3.5)	119 (1.9)	132 (2.2)	351 (5.7)

No. (%) of isolates with ceftriaxone MIC (µg/mL)									
	≤0.06	0.12	0.25	0.5	1	2	4	8	>8
Reference 1 (*n* = 200)	68 (34.0)	15 (7.5)	10 (5.0)	5 (2.5)	1 (0.5)	0 (0.0)	3 (1.5)	5 (2.5)	93 (46.5)
Sentry 2020^*a*^ (*n* = 6,128)	3,336 (54.5)	715 (11.7)	457 (7.5)	199 (3.2)	80 (1.3)	48 (0.8)	38 (0.6)	30 (0.5)	1,225 (20.0)

^*a*^ MIC distribution of enteric isolates in the SENTRY program collected from medical centers in Latin America and North America during 2020 (accessed from https://sentry-mvp.jmilabs.com/ on 11 March 2026).

Current guidance provided by the Infectious Diseases Society of America does not recommend piperacillin-tazobactam for treating extended spectrum β-lactamase Enterobacterales (ESBL-E) infections outside the urinary tract, even if the isolates test susceptible to piperacillin-tazobactam with current CLSI breakpoints (MIC ≤8 µg/mL) (5). Therefore, many clinical laboratories do not report piperacillin-tazobactam results for the ESBL-E isolates selected for testing in the Henderson and Sohani studies (6, 7). Meropenem susceptibility varied among the studies, with all of the isolates in the Henderson and Solani study susceptible compared to 75% of isolates in our study.

Tables 2 and 3 present the comparative VITEK 2-BMD piperacillin-tazobactam MIC data for all isolates, as well as for the subset of the ceftriaxone non-susceptible *Escherichia coli* and *Klebsiella pneumoniae* isolates included in our study to allow direct comparison with the formats used by Sohani et al. and Henderson et al.

**TABLE 2:** Piperacillin-tazobactam minimal inhibitory concentration (MIC) by broth microdilution (BMD) vs VITEK 2 MIC—Enterobacterales (Overall) using CLSI breakpoints^*a*^^,^^*b*^

	BMD	S	S	S	S	S	S	SDD	R	R	R
VITEK 2		0.25 µg/mL	0.5 µg/mL	1 µg/mL	2 µg/mL	4 µg/mL	8 µg/mL	16 µg/mL	32 µg/mL	64 µg/mL	>64 µg/mL
S	≤4 µg/mL	11	2	13	58	22	3	1			
S	8 µg/mL				1	5	1	3			
SDD	16 µg/mL					1	3	3			
R	32 µg/mL						1	3			2
R	64 µg/mL								1	1	
R	>64 µg/mL									1	46

Piperacillin-tazobactam—Enterobacterales (Overall)											
				Overall essential agreement	176/182	96.7%					
				Overall categorical agreement	170/182	93.4%					
				Minor errors	11/182	6.0%					
				Major errors	1/121	0.8%					
				Very major errors	0/51	0.0%					

^*a*^ Unclaimed performance (the manufacturer does not have FDA clearance to report the results with CLSI breakpoints).

^*b*^ Gray shading indicates the same MIC by BMD and Vitek 2.

**TABLE 3:** Piperacillin-tazobactam minimal inhibitory concentration (MIC) by broth microdilution (BMD) vs VITEK 2 MIC*—E. coli* & *K. pneumoniae* (ceftriaxone non-susceptible) using CLSI breakpoints^*a*^^,^^*b*^^,^^*c*^^,^^*d*^

	BMD	S	S	S	S	S	S	SDD	R	R	R
VITEK 2		0.25 µg/mL	0.5 µg/mL	1 µg/mL	2 µg/mL	4 µg/mL	8 µg/mL	16 µg/mL	32 µg/mL	64 µg/mL	>64 µg/mL
S	≤4			2	14	9	1				
	µg/mL										
S	8				1	3	1	2			
	µg/mL										
SDD	16						1				
	µg/mL										
R	32							2			2
	µg/mL										
R	64										
	µg/mL										
R	>64										31
	µg/mL										

Piperacillin-tazobactam*—E. coli* & *K. pneumoniae* (ceftriaxone non-susceptible)											
					Overall essential agreement	66/69	95.7%				
					Overall categorical agreement	64/69	92.8%				
					Minor errors	5/69	7.2%				
					Major errors	0/32	0.0%				
					Very major errors	0/33	0.0%				

^*a*^ Unclaimed performance (the manufacturer does not have FDA clearance to report the results with CLSI breakpoints).

^*b*^ Ceftriaxone non-susceptible based on BMD MIC.

^*c*^ Resistance genotypes: 7 AmpC, 23 carbapenem-resistant, 39 ESBL.

^*d*^ Gray shading indicates the same MIC by BMD and Vitek 2.

We hope the clarifications and supplemental data provided here contribute constructively to the ongoing discussion about piperacillin-tazobactam susceptibility testing in Enterobacterales.
